# Cardiac Signals Are Independently Associated with Temporal Discounting and Time Perception

**DOI:** 10.3389/fnbeh.2017.00001

**Published:** 2017-01-24

**Authors:** Bowen J. Fung, Damien L. Crone, Stefan Bode, Carsten Murawski

**Affiliations:** ^1^Melbourne School of Psychological Sciences, The University of MelbourneMelbourne, VIC, Australia; ^2^Department of Finance, The University of MelbourneMelbourne, VIC, Australia

**Keywords:** temporal discounting, time perception and timing, heart rate, interval timing, heart rate variability (HRV)

## Abstract

Cardiac signals reflect the function of the autonomic nervous system (ANS) and have previously been associated with a range of self-regulatory behaviors such as emotion regulation and memory recall. It is unknown whether cardiac signals may also be associated with self-regulation in the temporal domain, in particular impulsivity. We assessed both decision impulsivity (temporal discounting, TD) and time perception impulsivity (duration reproduction, DR) in 120 participants while they underwent electrocardiography in order to test whether cardiac signals were related to these two aspects of impulsivity. We found that over the entire period of task performance, individuals with higher heart rates had a tendency toward lower discount rates, supporting previous research that has associated sympathetic responses with decreased impulsivity. We also found that low-frequency components of heart rate variability (HRV) were associated with a less accurate perception of time, suggesting that time perception may be modulated by ANS function. Overall, these findings constitute preliminary evidence that autonomic function plays an important role in both decision impulsivity and time perception.

## Introduction

Cardiac signals have previously been associated with a wide range of psychopathologies, including substance abuse (Levin et al., 1992; Ingjaldsson et al., 2003), panic disorder and generalized anxiety disorders (Yeragani et al., 1993; Friedman and Thayer, 1998), obsessive compulsive disorder (Pittig et al., 2013), depression (Kemp et al., 2010), schizophrenia (Clamor et al., 2016) and post-traumatic stress disorder (Cohen et al., 1997). The link between cardiac signals and psychopathology is usually explained by the fact that these psychopathologies affect the function of the autonomic nervous system (ANS), which, in turn, directly impacts cardiac activity.

More recently, cardiac signals such as heart rate variability (HRV) have also been related to cognitive function in healthy individuals. For example, higher resting HRV has been associated with more adaptive attention to emotional stimuli (Park and Thayer, 2014), smaller startle responses (Ruiz Padial et al., 2003), better deliberate suppression of unwanted memories (Gillie et al., 2014) and thoughts (Gillie et al., 2015), more accurate and faster working memory retrieval (Hansen et al., 2003), and better self-control over dietary choices (Segerstrom and Nes, 2007). Furthermore, HRV has been shown to increase transiently during emotion regulation (Butler et al., 2006), and during difficult memory retrieval (Gianaros et al., 2004). Further to this, a direct causal relationship between ANS function and cognition has also been demonstrated via vagus nerve stimulation (for working memory processes; Clark et al., 1999).

Cardiac measurements have also been associated with performance in higher-level decision-making tasks. For example, individuals with low resting heart rates have been found to be more likely to make risky decisions (Schmidt et al., 2013). Transient heart rate, however, has been shown to slow directly after a loss in a gambling task, and this decrease in heart rate begins earlier (often prior to the outcome) in individuals who have better general performance in the task (Crone et al., 2004). It has also been shown that individuals with a higher power in the low frequency component of resting HRV generally perform better on gambling tasks (Drucaroff et al., 2011), and there is some evidence that direct vagus stimulation (which increases HRV) can enhance performance in these tasks (Martin et al., 2004). Furthermore, HRV (and its high frequency component) decreases during “stressful” unfair economic offers (such as those in the ultimatum game; Armin et al., 2011; Dulleck et al., 2014). These pressure-induced decreases in HRV have been shown to be more pronounced in individuals who cope less efficiently under high pressure situations (Laborde et al., 2014). While the reasons for these associations are not yet fully understood, these relationships demonstrate that in addition to their association with clinical disorders, cardiac signals are also associated with aspects of decision behaviors in healthy individuals.

One popular account, referred to as the neurovisceral integration model, posits that cardiac signals, and in particular HRV, index the state of a self-regulation network that spans across both the ANS and the central nervous system (Thayer and Lane, 2000, 2009). According to this model, the self-regulation network facilitates physiological, cognitive, and behavioral adaptability to environmental change. It predicts that a lack of such adaptability is associated with low HRV, whereas high HRV reflects a healthy, adaptive system.

The neurovisceral integration model places specific emphasis on the inhibitory role of the parasympathetic nervous system (PNS), the dysfunction of which results in prolonged and inappropriate physiological, emotional and behavioral responses (Thayer and Lane, 2000). As it has a relatively rapid influence on cardiac activity, one common method of estimating PNS function is via the high-frequency spectral component of HRV (HF-HRV; Berntson et al., 1997). Other studies have interpreted the low-frequency component of HRV (LF-HRV) as a reflection of sympathetic cardiac influence (Drucaroff et al., 2011; Dulleck et al., 2014), although this interpretation has been critisized (Reyes del Paso et al., 2013; see Discussion). The neurovisceral integration model also delineates neuroanatomical evidence for the relationship between cardiac signals and cognitive function. Specifically, this relies on the central autonomic network, which is responsible for central cardiac control, and comprises of the ventromedial prefrontal cortex (vmPFC), the central nucleus of the amygdala, anterior cingulate cortex, the insula, as well as several hypothalamic nuclei (Benarroch, 1993). Many of these areas have significant structural overlap with those that support the types of cognitive functions typically associated with cardiac signals (Thayer and Lane, 2000). In particular, cerebral blood flow in vmPFC has been correlated with changes in HRV induced by both emotional images (Lane et al., 2009) and working memory tasks (Gianaros et al., 2004). It has been suggested that vmPFC is the main locus of interaction between cognitive function and cardiac control (Thayer and Lane, 2000).

One fundamental aspect of self-regulation that has not previously been investigated from the perspective of cardiac physiology is regulatory behavior in the temporal domain, principally, impulsivity. While impulsivity is a multifaceted construct, two key aspects are of interest here: decision impulsivity, related to trading off immediate and delayed rewards, and impulsivity as it relates to time perception, which is required for appropriately timing actions (Evenden, 1999).

The main behavioral model of decision impulsivity is temporal discounting (TD; Bickel, 2015). It is typically characterized using a TD task, which assesses individuals’ choices between different magnitudes of reward available at different delays (Frederick et al., 2002). The rate of discounting (i.e., the rate of devaluation of reward per unit of time) has been shown to be relatively stable and heritable (Anokhin et al., 2015; Bickel, 2015), and has recently been proposed as an endophenotype (Bickel, 2015). Higher discount rates are apparent in multiple psychopathologies such as substance abuse, problem gambling, attention deficit hyperactive disorder, schizophrenia, depression and obesity (Bickel et al., 2012).

In the time perception domain, impulsivity is based on the notion that impulsive individuals overestimate, and hence under-reproduce time intervals (Wittmann and Paulus, 2008; Rubia et al., 2009; Moreira et al., 2016). For example, by using a duration reproduction (DR) task (in which participants reproduce a sample interval with a manual response), it has been shown that individuals with a impulsive personality traits tend to terminate their reproductions earlier than those without impulsive personality traits, as if they perceive the passage of time to be faster than it objectively is (van den Broek et al., 1992). Performance deficits in these types of time estimation tasks have been related to a range of psychiatric conditions: Parkinson’s disease, depression, bipolar disorder, schizophrenia, attention deficit hyperactivity disorder, autism, as well as anxiety disorders (Allman and Meck, 2012; Teixeira et al., 2013). Notably, there is considerable overlap in psychiatric disorders associated with abnormal cardiac signals, time perception and TD (Kemp et al., 2010; Allman and Meck, 2012; Bickel et al., 2012; Teixeira et al., 2013; Clamor et al., 2016).

Unlike TD, time perception has previously been investigated with respect to cardiac signals. For instance, one such study found that individuals with higher resting HRV were more accurate in a DR task (Pollatos et al., 2014). Another study found that individuals were more accurate in this task when they had a higher rate of heart rate slowing during the encoding of intervals (Meissner and Wittmann, 2011). Thus, there is some existing evidence that time perception accuracy and autonomic function are associated.

As the vmPFC is one of the primary regions involved in both central cardiac control and impulsivity (Kim and Lee, 2011), our first hypothesis was that specific aspects of cardiac signals are associated with behavioral performance during impulsivity-related tasks. Specifically, we hypothesized that HF-HRV (as an index of PNS function) would be associated with lower discount rates, and longer reproductions of time. Given that a common mechanism may underlie both decision impulsivity and time perception (Wittmann and Paulus, 2008; Moreira et al., 2016), and that damage to the vmPFC often results in both abnormal time perception and steeper TD (Berlin et al., 2004; Moreira et al., 2016), another hypothesis was that these two behavioral measures are related. To test these hypotheses, we measured cardiac activity while participants were completing a TD task and a DR task.

## Materials and Methods

### Participants

One hundred twenty healthy, right-handed participants (mean age 25, range 21–38, 63 female) from the general population were recruited via advertisement at The University of Melbourne. These individuals were primarily undergraduate students. Participants received AUD 15 for their participation. This study was approved by the University of Melbourne’s Human Research Ethics Committee (no. 1238359) and carried out in accordance with the Declaration of Helsinki. All participants gave informed written consent.

### Decision Impulsivity Task

Discount rates were measured using a TD task in which participants made binary choices between smaller amounts of money available at earlier times, and larger amounts of money available at later times. There is substantial evidence that the devaluation of reward over time follows a hyperbolic function of the form

(1) $$V=R.\frac{1}{1+kD}$$

where *V* is the subjective value of the reward, *R* is the objective reward amount received at delay *D*, and *k* is the discount rate (Mazur, 1987). Participants made a series of choices between immediately available amounts ranging from $20 to $30, and delayed (1, 2, 4, 6, 9 and 12 months) amounts that were chosen based on an algorithm constrained to estimate a threshold function that followed Equation 1 (Vul et al., 2010), as well as a softmax psychometric link function to map subjective values into choice probabilities (Miedl et al., 2012).

### Time Perception Task

Time perception was measured using a DR task in which, after being presented with a black square for a given duration, participants were asked to press and hold a response key to reproduce the duration (Zakay and Block, 1997). There were six durations ranging from 2 s to 15 s, spaced evenly on a logarithmic scale. We calculated the mean accuracy (reproduction minus sample interval), and mean coefficient of variation (CV; standard deviation divided by the sample interval; Gibbon, 1977) for each participant and each sample interval. Additionally, using least squares regression, we fitted a power function to each participant’s reproduced durations (Stevens and Galanter, 1957), of the form

(2) $$\mu (t)=\alpha (t{)}^{\beta }$$

where *μ(t)* is perceived duration and *t* is the sample duration. These parameters indicate differences in time perception between the encoding and the reproduction of an interval. The scale parameter α shifts the slope of the psychophysical function. Relative to unity, a smaller α parameter implies either a constantly accelerated perception of time during reproduction or a constantly decelerated perception of time during encoding. The exponent β models the shape of the psychophysical function: relative to unity, a smaller exponent implies concavity in the function (a decreasing slope), whereas a larger exponent implies convexity (an increasing slope). A smaller exponent could be interpreted as either an acceleration of perceived time during the reproduction of longer intervals, or a compression of time during the encoding of longer intervals. Ultimately, deviations from unity for both of these parameters indicates deviation from veridical time and thus inaccuracy in time perception. Both of these parameters were used for statistical analyses. Compared to a logarithmic Weber-Fechner law (α log(*t + c*); Grondin, 2001), the fit of the data to Equation 2 had a larger *R*^2^ (mean 0.83) for 75 participants (80% of cases).

### Equipment and Physiological Recording

The Psychophysics Toolbox (Brainard, 1997) running on MATLAB 8.4 was used for stimulus presentation, and a RB-540 Cedrus button box was used to capture responses.

Electrocardiogram (ECG) was measured using two amplified adhesive Ag/AgCl EEG electrodes in a modified Lead II Einthoven configuration: one positioned under the right clavicle and one above the left side of the third rib, as well as two implicit reference electrodes positioned underneath the left clavicle. These electrodes interfaced with a BioSemi ActiveTwo system running ActiView acquisition software, and recorded at a sampling rate of 512 Hz. Data were recorded for the entirety of the experimental session (approximately 1 h).

### Experimental Procedure

**Figure 1:**
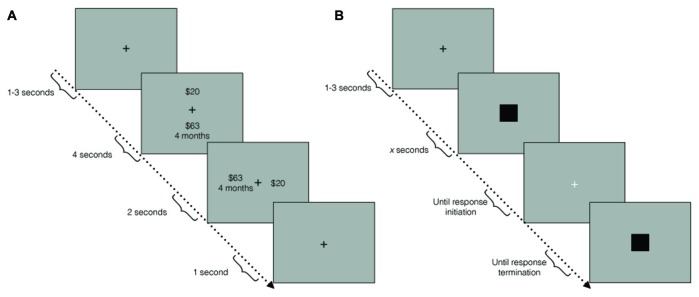
**(A)** Temporal discounting (TD) paradigm schematic. In each trial, a fixation cross first appeared for 1 s–3 s. Subsequently, two choice options were displayed, one above the other, for 4 s. During the response window, the choice options were displayed side by side, for 2 s. A fixation cross then appeared for an inter-trial interval of 1 s. **(B)** Duration reproduction (DR) paradigm schematic. In each trial, a fixation cross first appeared for 1 s–3 s. Subsequently, a black square was displayed for a pseudo-randomly chosen duration (the sample interval). A white fixation cross then signaled the response window. Once participants initiated a response, another black square was presented until participants terminated their response (the reproduced interval).

After preparing the ECG, participants were asked to complete a block of the TD task (Figure 1A). In each trial, a fixation cross appeared for a duration drawn from a uniform distribution on the interval from 1 s to 3 s. Subsequently, two choice options were presented on the screen, one above the other, for 4 s (e.g., “$19.02 in 2 months” and “$10 today”). On the following screen, the two options were then presented side by side for 2 s. Screen positions of the choice options (top/bottom, left/right) were counterbalanced throughout the experimental session. Participants chose one of the two options by pressing one of two buttons on a response pad. After an option was selected, a fixation cross was displayed for a 1 s inter-trial interval. The task terminated when the threshold estimation algorithm converged, or the task reached 80 trials. Participants were explicitly instructed to treat each trial as independent. To establish incentive compatibility, participants were told that a dice would be rolled at the end of the experiment for a chance to win the choice made in one of the trials.

Participants then completed the DR task (Figure 1B). In each trial, participants were first presented with a black fixation cross for between 1 s and 3 s, randomly drawn from a uniform distribution. This was followed by a black square, which was presented for one of six pseudo-randomly chosen intervals (see Time Perception Task). A white fixation cross then appeared until participants initiated a response to reproduce the interval. Once a response was initiated, an identical black square was presented until termination of the response. The next trial began immediately after response termination. There were five repetitions of each interval for a total of 30 trials. Participants were instructed to avoid chronometric counting, to mitigate the effect of sub-vocal rhythm strategies, which can improve accuracy artificially (Rattat and Droit-Volet, 2011).

Participants then completed a second block of the TD task, identical to the first. The purpose of splitting the TD task over two blocks was to test the effect of a feedback manipulation (reporting overestimation vs. underestimation) after the DR task on the second TD task. These results are not reported in this article as we found no effects on performance between conditions (see below for control analyses).

Finally, participants completed a series of questionnaires, administered via web-browser, that were used to assess adherence to task instructions (not reported here in detail). After completion of all experimental tasks, participants were debriefed. A dice was rolled to determine whether they won extra money from the TD task. If a participant won, one trial from the TD was selected, and the participant was paid the chosen amount of money at the chosen delay.

### ECG Protocol

**Figure 2:**
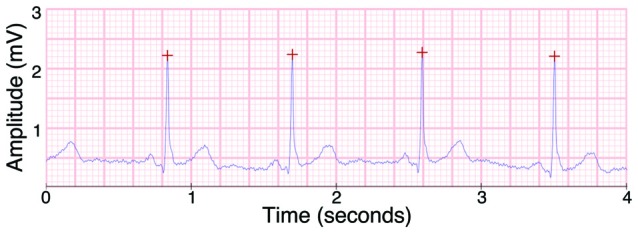
**Example of the electrocardiogram (ECG) signal as recorded by the BioSemi ActiveTwo EEG system, with R peaks as detected by Kubios heart rate variability (HRV) marked with red crosses**.

In order to estimate general cardiac parameters for a sufficiently long period of time, ECG was recorded over the entirety of the experimental session. Data were detrended and underwent automatic artifact correction using Kubios HRV software (Tarvainen et al., 2014). Any remaining artifacts were manually removed after visual inspection. The software automatically detected R-wave peaks, and any incorrectly identified peaks were manually removed. Heart rate was calculated as the total number of R-wave peaks divided by the total recording time in minutes. We used rMSDD (square root of the mean squared differences of successive R-R intervals) as a measure of HRV. An exemplar ECG indicating automatically identified R-wave peaks is shown in Figure 2.

We further decomposed HRV into component frequency domains using a Fast-Fourier transform. Power in the high frequency (HF-HRV; 0.15–0.4 Hz) band is widely believed to reflect HRV responses to parasympathetic inputs (Berntson et al., 1997), but is also affected by respiratory patterns (Grossman and Kollai, 1993). Power in the low frequency (LF-HRV; 0.04–0.15 Hz) band has been assumed to reflect changes of sympathetic origin (Berntson et al., 1997), although this assumption has been contested (Reyes del Paso et al., 2013; see Discussion). Note that our approach did not allow us to measure fluctuations in cardiac activity between task phases, but we aimed to obtain a reliable estimate of general and sustained activity across time while participants were engaged in the tasks. Therefore, these measures constituted a combination of general cardiac activity during task performance and the short rest periods for each individual, while neglecting task-phase specific fluctuations.

### Data Analysis

We excluded participants who chose an option with a negative monetary amount in the TD task more than once (3 participants excluded), missed more than 10 responses in the TD task (10 participants excluded) or whose mean responses in the DR task were more than 3 s away from the sample average (3 participants excluded). Note that the above constituted attentional criteria only, and we did not exclude participants based on impulsivity measures. This makes it unlikely that the retained participants differed in impulsivity from the excluded ones, who showed strong a lack of attention to the task. One additional participant failed to complete the experiment. In addition, we excluded another 17 participants due to inability to calculate cardiac measures due to poor ECG quality. All analyses were performed on data from the remaining participants for which we had complete data sets (94 participants).

Discount rate, time perception parameters and cardiac variables were non-normally distributed. Specifically, discount rates were right-skewed, the scale parameters from the DR psychophysical function were right-skewed, while the exponents were left skewed, and HRV and both frequency components of HRV were heavily right-skewed. We therefore used non-parametric analyses where appropriate. We used a paired Wilcoxon signed-rank test to identify whether there were any differences in discount rate between each block of the TD task. Kruskal-Wallis tests were used to assess the main effects of sample interval on DR task measures. Spearman correlations were used to explore relationships between: (a) DR measures; (b) discount parameters; and (c) ECG measures.

For all statistical tests, the significance level was set to *p* < 0.05. Multiple comparisons corrections were carried out for each set of correlational analyses (a, b and c, above) using the Holm-Bonferroni method (Holm, 1979). All statistical analyses were performed using R, version 3.2.1.

## Results

### Temporal Discounting

First, we established whether data from both TD blocks could be pooled for the following analyses. The median discount rate *k* for the first and second blocks of the TD task were 0.07 (range = 0.01–0.12) and 0.07 (range = 0.01–0.22), respectively. A Wilcoxon signed-rank test did not reveal significant differences between *k*, measured in the two blocks (*T* = 0.50, *p* = 0.619). Thus, for subsequent analyses we used the participant mean of *k* in the two blocks.

### Duration Reproduction

Reproduced durations exhibited several characteristics commonly seen in temporal reproduction data. Reproduced durations increased monotonically with the sample interval (*H*_(5)_ = 746.74, *p* < 0.001), implying that participants followed instructions. Moreover, longer sample intervals were systematically under-reproduced (sample interval on accuracy; *H*_(5)_ = 380.21, *p* < 0.001). We quantified this effect with quantile regression (median): for every second increase in the sample interval, there was a 0.25 (SD = 0.01) second decrease in the reproduced duration, relative to a perfect reproduction (*p* < 0.001).

We also found an increase in response variability with longer sample intervals (*H*_(5)_ = 407.62, *p* < 0.001). According to scalar property, timing variability scales proportionally with the interval to be timed (Gibbon, 1977). To test whether the scalar property held for our data, we computed the CV (the standard deviation of the estimation divided by the mean estimation). The CV was significantly affected by the sample interval (*H*_(5)_ = 53.05, *p* < 0.001), in line with other recent research that has reported violations of the scalar property (Wearden and Lejeune, 2008; Lewis and Miall, 2009). This change in CV was well described by a simple logarithmic regression with a slope of −0.042 (*p* = 0.024, *R*^2^ = 0.76, SEM = 0.01), coinciding with data from previous studies (Lewis and Miall, 2009).

We then fitted a psychophysical function (Equation 2) to the reproduction data. The mean estimated value of the scale parameter *α* was 1.035 (SD = 0.42). It was not significantly different from unity (*t*_(93)_ = 0.80, *p* = 0.426), suggesting that, on average, there was no constant deviation from verdical time. The mean exponent *β* was estimated at 0.899 (SD = 0.17). It was significantly different from unity (*t*_(93)_ = −5.37, *p* < 0.001), suggesting that participants psychophysical functions were concave, corroborating the increasing underestimation with larger sample intervals noted in previous literature (Lewis and Miall, 2009). Within participants, the scale and exponent parameters were strongly negatively correlated (*r* = −0.87, *p* < 0.001), while the exponent and CV were strongly positively correlated (*r* = 0.93, *p* < 0.001; Table 1).

**Table 1:** **Correlations between behavioral and cardiac measures (*N* = 94)**.

	*k*	Accuracy	CV	Scale	Exponent	HR	RMSSD	HF-HRV
Accuracy	0.17
CV	−0.12	0.06
Scale (*α*)	0.10	0.23**	−0.87***
Exponent (*β*)	−0.03	0.21*	0.93***	−0.87***
HR	−0.23*	−0.05	−0.13	0.05	−0.10
RMSSD	0.11	0.05	−0.05	0.16	−0.15	−0.37***
HF-HRV	−0.06	0.01	−0.09	0.20	−0.22*	−0.25**	0.89***
LF-HRV	0.15	−0.08	−0.20	0.28**	−0.31**	−0.36***	0.71***	0.64***

Note: reported p-values are uncorrected *p < 0.05; **p < 0.01; ***p < 0.001. *k, discount rate; CV, coefficient of variation; HR, heart rate; RMSSD, Square root of the mean squared differences between successive RR intervals; HF-HRV, high frequency HRV power; LF-HRV, low frequency HRV power*.

### Cardiac Function

First, we confirmed that there were no significant differences in physiological measurements between feedback conditions using one-way Kruskal-Wallis rank sum tests (see above). There were no significant differences for mean heart rate (*H*_(3)_ = 4.46, *p* = 0.22), rMSSD (*H*_(3)_ = 0.868, *p* = 0.833, HF-HRV (*H*_(3)_ = 1.76, *p* = 0.624, or LF-HRV (*H*_(3)_ = 4.61, *p* = 0.203). Additional *post hoc* pairwise Nemenyi tests also showed no significant differences between any conditions. Hence, we concluded that the additional feedback manipulation had neither an effect of behavior nor on cardiac function, allowing us to use the cardiac measures here as stable estimates for sustained cardiac activity across the experiment.

The mean heart rate across participants was 84.91 bpm (SD = 15.55). The mean rMSSD across participants was 36.06 ms (SD = 16.57). The mean HF-HRV across participants was 621.71 ms^2^ (SD = 623.38). The mean LF-HRV across participants was 1145.39 ms^2^ (SD = 846.96). All measures of HRV were negatively correlated with mean heart rate (all *p* < 0.001, except for HR and HF-HRV, *p* = 0.02, see Table 1 for correlation coefficients), and positively correlated with each other (all *p* < 0.001, see Table 1 for correlation coefficients).

### The Relationship between Temporal Discounting and Cardiac Function

**Figure 3:**
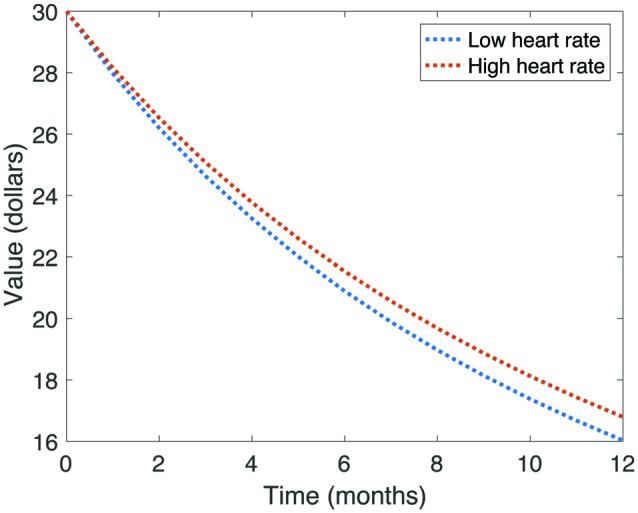
**Illustration of average discount function of value over time, split by heart rate.** Note that those participants with higher heart rates had shallower discount functions.

We first tested the relationship between TD and cardiac function. To do so, we calculated Spearman rank correlations between the discount rate k and mean heart rate, rMSSD, as well as LF-HRV and HF-HRV components. All test statistics are reported in Table 1. We found a significant negative correlation between discount rate and mean heart rate (*r* = −0.23, *p* = 0.032). However, this relationship did not survive correction for multiple comparisons (*p* = 0.108). To illustrate the relationship between mean heart rate and discount rate, we divided our sample around the median heart rate value, and plotted hyperbolic discount functions for each group over a 12-month period (Figure 3). Note that individuals with higher heart rates had a shallower discount function.

### The Relationship between Duration Reproduction and Cardiac Function

We then tested the relationship between DR and cardiac function. We calculated Spearman rank correlations between reproduction measures (mean accuracy, CV, and psychophysical scale (*α*) and exponent (*β*) parameters), and between mean heart rate, rMSSD, as well as LF-HRV and HF-HRV components. All uncorrected test statistics are reported in Table 1. We found significant negative correlations between HF-HRV and the exponent *β* of the reproduction function (*r* = −0.22, *p* = 0.039), as well as LF-HRV and the exponent (*r* = −0.31, *p* = 0.004), suggesting that individuals with higher power in both HRV frequency components had a relatively more concave psychophysical function. Because HF-HRV more closely indexes cardiac parasympathetic tone when heart period is taken into account (Grossman and Kollai, 1993), we used a Spearman semi-partial correlation to reanalyze the relationship between HF-HRV and the exponent, while controlling for heart period. The relationship between these two variables remained significant (*r* = −0.26, *p* = 0.014). After correction for multiple comparisons, the relationship between LF-HRV and the exponent remained significant (*p* = 0.02), but the relationship between HF-HRV and the exponent closely missed the critical threshold (*p* = 0.056).

**Figure 4:**
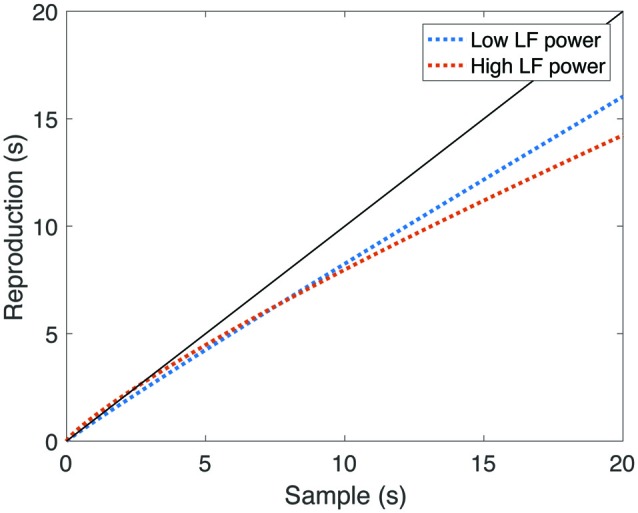
**Reproduced durations as a function of sample interval, split by low-frequency component of HRV (LF-HRV) power.** The black line represents veridical time perception. Note that in those participants with high LF-HRV, short intervals were relatively overproduced, and longer intervals were relatively under-produced, while in those participants with low LF-HRV, longer reproduced durations were more accurate.

We also found a significant positive correlation between LF-HRV and the scale parameter *α* (*r* = 0.28, *p* = 0.009), suggesting that individuals with higher LF-HRV power either perceived time as either relatively fast during the encoding of the interval, or relatively slow during reproduction. This relationship remained significant after correction for multiple comparisons (*p* = 0.043). To illustrate the relationship between LF-HRV and DR, we divided our sample around the median LF-HRV value, and plotted mean reproduced duration as a function of sample interval (Figure 4). Note that in those with high LF-HRV, short intervals were slightly overproduced, and longer intervals are substantially underproduced, while in those with low LF-HRV, reproduced durations were more accurate. Overall, these findings supported our hypothesis of a relationship between time perception and cardiac function.

### The Relationship between Temporal Discounting and Duration Reproduction

Finally, we tested the relationship between TD and DR. We calculated Spearman rank correlations between DR measures (mean accuracy, standard deviation, CV and psychophysical scale and exponent parameters), and the discount rate *k*. No significant correlations were found. All test statistics are reported in Table 1.

## Discussion

In order to investigate the relationship between time perception, decision impulsivity and cardiac signals, we assessed cardiac activity of individuals while they performed DR and TD tasks. We found a negative correlation between the mean heart rate and discount rate, suggesting that individuals with higher heart rates were more patient. We also found positive correlations between LF-HRV and the parameters of the psychophysical function for DR, suggesting that those with higher LF-HRV power perceived time differently during the encoding and reproduction of the interval, and had poorer sensitivity to longer intervals. Additionally, we found a positive correlation between HF-HRV and the exponent parameter of the psychophysical function, suggesting that those with high HF-HRV power increasingly underestimated longer intervals.

TD has previously been related to other physiological measures, such as pupil dilation (Lempert et al., 2016), but to our knowledge, our study is the first to relate it to cardiac signals. The neurovisceral integration model (Thayer and Lane, 2000, 2009) is built on the notion that greater regulatory control is associated with greater inhibitory parasympathetic functionality. Thus, it would predict that discount rate was negatively associated with HRV. Here, we observed that healthy individuals with higher heart rates had lower discount rates, indicating lower impulsivity, which does not support this prediction.

However, recent work has shown that during TD tasks, choices toward delayed rewards are more likely when sympathetic responses (measured via pupil dilation) to these options are greater (Lempert et al., 2016). As heart rate is primarily mediated by sympathetic activity, it is possible that the observed association between heart rate and discount rate constitutes a similar phenomenon: individuals with lower discount rates were also the ones with higher sympathetic responses, which reflected their tendency to choose delayed rewards. In support of this interpretation, previous studies have shown that higher levels of impulsivity (measured using personality questionnaires) were associated with lower resting heart rates (Mathias and Stanford, 2003) and with lower heart rates during the preparation of an independent task (Allen et al., 2009). Thus our findings provide further evidence that sympathetic nervous system responses may be associated with decision impulsivity.

We also observed a correlation between the parameters of the psychophysical function for DR and both LF- and HF-HRV. It is generally accepted that HF-HRV reflects PNS cardiac influence (Berntson et al., 1997), although it has also been reported that this can be confounded by respiratory patterns (Grossman and Kollai, 1993). While we did not directly measure respiratory activity in this study, an analysis controlling for this possible influence (Grossman and Kollai, 1993) did not alter our results. It has also been noted that interindividual associations between HF-HRV and parasympathetic cardiac influence are modest (Grossman and Taylor, 2007). Thus, while these findings appear to indicate that individuals with higher parasympathetic cardiac influence had poorer sensitivity to longer intervals, some caution should be taken in interpreting our results solely along these lines.

The interpretation of LF-HRV is more contentious than that of HF-HRV. In the psychological literature, increased LF-HRV has previously been related to fatigue and attentional deficits (Egelund, 1982; Mascord and Heath, 1992; Fairclough and Graham, 1999). This may provide an explanation of our findings, as lowered attentional capacity may lead to shorter encoded durations (Zakay and Block, 1995), or stronger temporal context effects and a regression to the mean of the sample durations (Jazayeri and Shadlen, 2010).

The physiological source of LF-HRV is highly contentious. It has been argued that differences in LF-HRV are due to either gross sympathetic activation (Malliani et al., 1991), a combination of parasympathetic and sympathetic influences (Reyes del Paso et al., 2013), baroreflex function (changes in HR due to changes in blood pressure; Goldstein et al., 2011), or the output of a central, LF oscillator (Cooley et al., 1998). On one hand, there is existing support for the possibility that the relationship between LF-HRV and time perception is sympathetically mediated. Time perception has been reported to “accelerate” relative to objective time during life-threatening situations (Stetson et al., 2007), under conditions of high body temperature (Wearden and Penton-Voak, 1995), as well as during general emotional arousal (Gil and Droit-Volet, 2012), which corresponds to sympathetic physiological changes (Thompson, 2013). Likewise, previous studies have shown that another measure of high sympathetic activity (cardiac pre-ejection period) is associated with lower temporal sensitivity (Cellini et al., 2015). On the other hand, the positive correlations among LF-HRV, HF-HRV and rMSSD (as well as the negative correlation between HF-HRV and the exponent parameter of temporal reproduction), suggest that our measurement of LF-HRV may represent predominantly parasympathetic influence. However, if this is the case, then our results directly contradict previous findings showing that parasympathetic function and vagal control are associated with increased time perception accuracy, rather than the decrease in accuracy we observed in those with high LF-HRV (Cellini et al., 2015). One possible resolution of this discrepancy is that this previous study used different time scales (around 1 s) and different timing mechanisms may be recruited for different durations (Ivry and Spencer, 2004). This interpretation is also inconsistent with the relationships between HRV, working memory and time perception, as higher vagal control has been associated with better working memory (Hansen et al., 2003), which is, in turn, associated with increased time perception accuracy (Broadway and Engle, 2011). Further to this, we did not observe any relationships between time perception and HRV in the time domain (rMSSD). Thus, it is difficult to comment on the physiological source of the observed result. Future studies could clarify this by employing more interpretable measures of SNS activity, such as skin conductance level (Mella et al., 2011), or levels of neurotransmitters such as noradrenaline (Zygmunt and Stanczyk, 2010). However, our results are consistent with the psychological attention-based interpretation of LF-HRV, and more broadly the associations between HRV frequency components and time perception measures support previous suggestions that periodic internal signals may constitute a time-keeping mechanism (Craig, 2009; Wittmann, 2015).

It is important to note that some of the observed correlations (e.g., between discount rates and mean heart rate) were only significant before correction for multiple comparisons. The uncorrected results are nevertheless interesting, given the absence of existing literature on this topic, and the exploratory nature of our study. We emphasize that these findings require further investigation.

Some limitations of the current study could be addressed in future work. For example, in addition to using directly interpretable measures of SNS activity, future studies could also employ other non-invasive physiological measures, such as eyeblink rate or pupillometry. On the other hand, an assessment of action, as opposed to choice impulsivity (such as a Go-Nogo task), or personality traits, may also be desirable (Glicksohn et al., 2006). It would also be of interest to investigate whether the observed correlations between cardiac measures and time perception would extend to other time estimation paradigms, such as a temporal bisection task, or shorter or longer intervals. Finally, this line of inquiry may be of potential utility in the diagnosis and treatment of psychopathologies that involve impulsivity, such as substance abuse, problem gambling, attention deficit hyperactive disorder, schizophrenia, depression and obesity (Bickel et al., 2012).

We note that as our physiological measures were not isolated to the presentation of stimuli, we were not able to determine whether the observed correlations were driven by task-related responses or trait-like autonomic function. Further studies with baseline measures, longer recordings, and more isolated task phases may be able to address this question, as well as whether the observed relationships were mediated by other personality traits.

While we found that cardiac signals were independently correlated with both TD and DR, we did not find any direct relationship between these two components of impulsivity (Wittmann and Paulus, 2008; Moreira et al., 2016). One possible reason for this is the difference in temporal scale (months in TD vs. seconds in DR), which may recruit different cognitive mechanisms. Future research could address this by using a discounting task with time delays more similar to those used in time perception tasks.

In conclusion, our study shows that differences in ANS function may help to explain inter-individual heterogeneity in both TD and time perception. The association between TD and heart rate supports the notion that low arousal might be related to higher impulsivity, similar to previous perspectives on trait impulsivity (Eysenck, 1993) and previous research using alternate measures (Mathias and Stanford, 2003; Allen et al., 2009; Lempert et al., 2016). Given the conflicting interpretations of cardiac indices, our results concerning DR are difficult to interpret from a physiological perspective. Psychologically, however, our results appear to reflect an association mediated by differences in attention. We found no evidence for a relationship between TD and time perception, which reinforces the idea that these measure different aspects of impulsivity, which appears to be a rather complex construct (Evenden, 1999). Our findings further show that ANS function could provide distinct indices for such aspects of impulsivity, opening up new avenues for future research to decompose impulsivity beyond TD and time perception (e.g., response inhibition; Krypotos et al., 2011).

## Author Contributions

BJF and DLC contributed to the design of the study, collection and analysis of the data, and drafting of the manuscript. SB and CM contributed to the design of the study, statistical analysis, and drafting of the manuscript. All authors gave final approval for publication.

## Conflict of Interest Statement

The authors declare that the research was conducted in the absence of any commercial or financial relationships that could be construed as a potential conflict of interest.
